# Effect of Cold Rolling Reduction Rate on the Microstructure and Properties of Q&P Steel with a Ferrite-Pearlite Initial Structure

**DOI:** 10.3390/ma16186102

**Published:** 2023-09-07

**Authors:** Shengwei Wang, Mengxiao Chen, Mingyue Yang, Yuhe Huang, Shuize Wang, Xinping Mao

**Affiliations:** 1 Institute for Carbon Neutrality, University of Science and Technology Beijing, Beijing 100083, China; 2 Shanghai Key Laboratory of Materials Laser Processing and Modification, School of Materials Science and Engineering, Shanghai Jiao Tong University, Shanghai 200240, China; 3 Research Institute, Baoshan Iron and Steel Co., Ltd., Shanghai 201999, China; 4 Institute of Steel Sustainable Technology, Liaoning Academy of Materials, Shenyang 110000, China

**Keywords:** Q&P steel, microstructure and properties, cold rolling reduction rate, TSCR, retained austenite, phase transition

## Abstract

Quenching and partitioning (Q&P) steel has garnered attention as a promising third-generation automotive steel. While the conventional production (CP) method for Q&P steel involves a significant cumulative cold rolling reduction rate (CRRR) of 60–70%, the thin slab casting and rolling (TSCR) process has emerged as a potential alternative to reduce or eliminate the need for cold rolling, characterized with a streamline production chain, high-energy efficiency, mitigated CO_2_ emission and economical cost. However, the effect of the CRRR on the microstructure and properties of Q&P steel with an initial ferrite-pearlite microstructure has been overlooked, preventing the extensive application of TSCR in producing Q&P steel. In this work, investigations involving different degrees of CRRRs reveal a direct relationship between increased reduction and decreased yield strength and plasticity. Notably, changes in the microstructure were observed, including reduced size and proportion of martensite blocks, increased ferrite proportion and decreased retained austenite content. The decrease in yield strength was primarily attributed to the increased proportion of the softer ferrite phase, while the reduction in plasticity was primarily linked to the decrease in retained austenite content. This study provides valuable insights for optimizing the TSCR process of Q&P steel, facilitating its wider adoption in the automotive sector.

## 1. Introduction

The pursuit of lightweight, high-strength and high-toughness steel for automobile applications has become increasingly crucial in light of the energy crisis and environmental concerns. Advanced high-strength steel offers the potential to reduce weight, enhance energy efficiency and maintain safety in automobile bodies [1,2]. Meeting the demands for lightweight construction and safety requires advanced high-strength steel with a combination of exceptional strength and ductility. Quenching and partitioning (Q&P) steel [3,4,5,6], as a third-generation advanced high-strength steel, possesses the ability to fulfill both requirements concurrently [7]. The Q&P process [3,5,8,9] involves either partial or full austenitization, followed by quenching to a temperature between the martensite start temperature (Ms) and martensite finish temperature (Mf), in order to obtain a specific fraction of martensite. The subsequent isothermal treatment, known as “partitioning”, occurs either at the quenching temperature (one-step Q&P) or at a higher temperature (two-step Q&P), which allows for carbon redistribution from the quenched martensite to untransformed austenite. The addition of elements, such as Al, Si, or P, prevents the formation of cementite during partitioning [10]. Consequently, the carbon-enriched austenite is stabilized at room temperature after the final cooling. Meanwhile, the stabilized austenite is maintained in a metastable state, enabling deformation induced phase transformation to be activated during straining. Therefore, the effect of Q&P treatment on mechanical properties is highly dependent on the activation of the transformation-induced plasticity (TRIP) effect, which is determined by the volume fraction and stability of the retained austenite (RA) [11,12,13].

In the conventional production process of Q&P steel, hot rolling, cold rolling and Q&P heat treatment are employed [14]. Cold rolling with a reduction rate (ratio of the reduction in plate thickness to the initial thickness of the plate) of 60–70% is necessary [15], as the typical required thickness for Q&P steel in service is considerably thinner (0.7~1.2 mm) than that of conventional hot-rolled plates (2~3 mm). Meanwhile, in order to reduce the cold rolling force, the hot rolling-coiling temperature must be controlled to adjust the microstructure of the hot-rolled plate to a softer ferrite-pearlite structure [16]. To address the global focus on carbon reduction, especially in steel industry, the thin slab casting and rolling (TSCR) technology is being widely promoted due to its environmentally friendly and energy-saving characteristics [17]. TSCR technology enables the production of thinner hot-rolled plates with improved temperature uniformity; thus, reducing or even eliminating the need for cold rolling [18].

The process of cold rolling significantly affects the microstructure and properties of Q&P steel. As the CRRR increases, together with the elongation of ferrite and fracture of pearlite, the density of dislocations and stored deformation energy also increases [19]. The degree of deformation, defect density and stored strain energy impact the recrystallization of ferrite, as well as the nucleation and growth kinetics of austenite in accompanying heating; thus, influencing the distribution and morphology of ferrite and austenite before quenching [20]. Such an effect on the morphology and distribution of austenite plays a crucial role in carbon partitioning, affecting the stability of associated retained austenite in Q&P steel [21,22].

Previous research on Q&P steel has often overlooked the impact of cold rolling due to the processes’ limitations. The effectiveness of tunning cold rolling parameters in tailoring the morphology and distribution of austenite further influences the mechanical property of Q&P steel, and there is a pressing need to systematically investigate the influence of different CRRRs on the microstructure and properties of Q&P steel with a ferrite-pearlite microstructure in the hot-rolled state. In this study, we aim to address this research gap and provide comprehensive insights into the effects of CRRRs on Q&P steel. By elucidating the relationship between cold rolling, microstructure and properties, we strive to enhance the understanding of Q&P steel manufacturing processes and optimize its performance for automotive applications.

## 2. Materials and Methods

**Figure 1:**
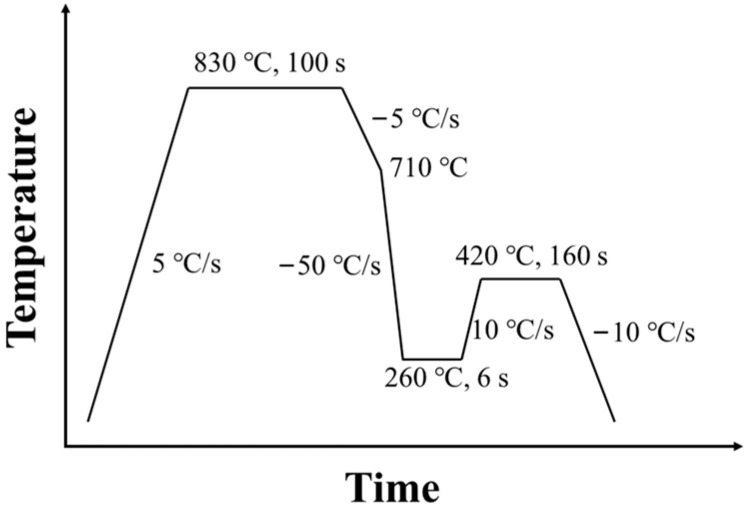
The schematic diagram of Q&P heat treatment.

The steel utilized in this study exhibited a chemical composition (wt.%) consisting of C-0.20, Si-1.75, Mn-2.00, with Fe serving as the balancing component. The thickness of the hot-rolled plate billet was 55 mm, which was rolled to 1.8 mm through a 5 passes hot rolling, and then cold rolled through different passes to get the final sample with a different reduction percentage. Cold rolling was performed at reduction rates of 10%, 40% and 70%; thus, creating varying thickness in the steel plates. To conduct Q&P treatments, the CCT-AY-II heat treatment system for thin steel sheets was employed with a specimen width of 70 mm and length of 220 mm. The schematic diagram of Q&P heat treatment is shown in Figure 1. The samples underwent a specific heating and cooling cycle, starting with heating to 830 °C at a rate of 5 °C/s for 100 s to achieve partial austenitization. Subsequently, they were rapidly cooled to 710 °C at a rate of 5 °C/s, followed by further cooling at 50 °C/s to 260 °C for 6 s. To facilitate partitioning, the samples were re-heated and maintained isothermally at 420 °C (partitioning temperature) for 160 s before undergoing quenching at a rate of −10 °C/s to room temperature.

The microstructures of the samples were examined using a GeminiSEM500 field emission scanning electron microscope (SEM) operating at 15 kV. Prior to analysis, metallographic samples were wire-cut, ground, polished and etched with 4% nital. To perform EBSD measurements, a PHI710 auger electron spectrometer with an EBSD detector was utilized, with an accelerating voltage of 20 kV, a step size of 50 nm, and a tilt angle of 70°. X-ray diffraction (XRD) experiments were carried out using a Bruker D8 Advance diffractometer operating at 40 kV and a current of 150 mA, with Cu Kα radiation. The samples for both EBSD and XRD analyses were taken by wire cutting with a speed of 0.5 mm/min. Then, the samples were ground using a polisher (ground to #400~#2000), and polished by using 3 μm and 1 μm diamond paste. To achieve the desired surface finish, the samples were finally polished and etched in an electrolyte of 10% perchloric acid and 90% ethanol with an applied voltage of 20 V over a duration of 30 s. To calculate the amount of retained austenite (RA), the (200), (220) and (311) austenite peaks, as well as the (200) and (211) ferrite peaks were considered. The volume fraction of retained austenite (*V_γ_*) was determined using the following equation [2,23,24]:

*V_γ_* = 1/(1 + G(*I_α_/I_γ_*)), (1)

where *I_α_* and *I_γ_* are the integrated intensity of the bcc and fcc phases, respectively, and the G value for each combination is referred to Ref. [23]. The volume fraction of austenite is the average of the six *V_γ_* values. EBSD is limited by the step size and resolution and is unable to resolve retained austenite at the nanometer scale; thus, the volume fraction of retained austenite measured will be less than that measured by XRD. Three parallel specimens of each sample were tested, mechanical properties and the calculated retained austenite volume fraction were averaged arithmetically. To measure the tensile properties of the samples with different morphological characteristics of microstructures, uniaxial tensile tests were conducted using standard specimens (according to the GB/T 228.1-2010 standard, Ref. [25]) with a gauge length of 50 mm. The tensile direction was parallel to the rolling direction. The mechanical properties were measured at room temperature on an INSTRON 5581 tensile testing machine with a crosshead displacement of 2 mm/min. Three parallel specimens of each sample were tested, and the mechanical properties and the calculated retained austenite volume fractions were averaged arithmetically. Heat treatments were performed on a DIL-805 A/D type dilatometer to test the phase-transition temperature and the evolution of microstructures during the Q&P process.

## 3. Results

### 3.1. Initial Microstructures before the Q&P Treatment

**Figure 2:**
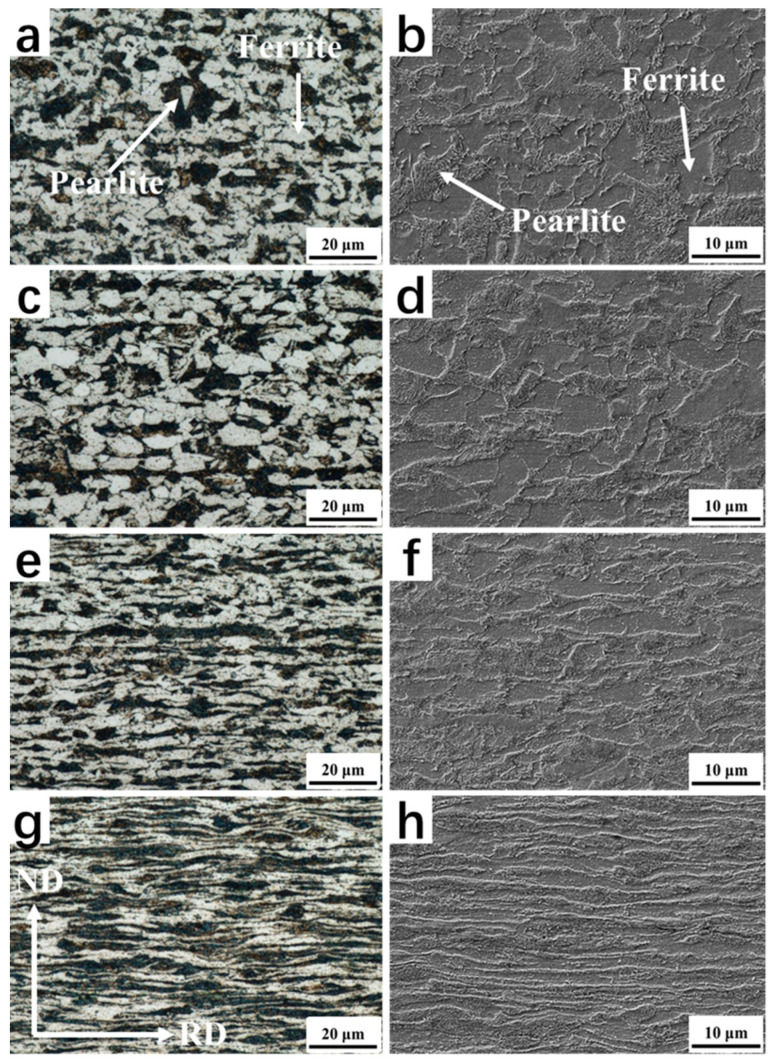
OM and SEM micrographs of initial microstructures before the Q&P treatment. (**a**,**b**) The original microstructure of hot-rolled plate produced by thin slab casting and rolling (TSCR) process. (**c**–**h**) The microstructure morphology of 10%, 40% and 70% cold rolling reduction rates, respectively.

Figure 2 illustrates the optical microscopy (OM) and scanning electron microscopy (SEM) micrographs of the hot-rolled plate and the cold-rolled plate at different reduction rates. The coiling temperature was precisely controlled at 600 °C, which falls within the pearlite transformation range. As depicted in Figure 2a,b, the hot-rolled plate, following coiling, exhibited a characteristic ferrite-pearlite microstructure. The ferrite grains displayed a polygonal shape, and the volume fraction of pearlite was estimated to be approximately 35%. Upon subjecting the material to cold rolling at varying reduction rates, distinct changes in the microstructure were observed (Figure 2c–h). These changes included differing levels of pearlite fragmentation and ferrite elongation. Notably, at a reduction rate of 70%, a prominent banded structure emerged along the rolling direction (Figure 2g,h). These findings suggest that the microstructural evolution of the plate is strongly influenced by the extent of the cold rolling reduction and underscore the importance of careful control over this process parameter.

### 3.2. Final Microstructures after the Q&P Treatment

**Figure 3:**
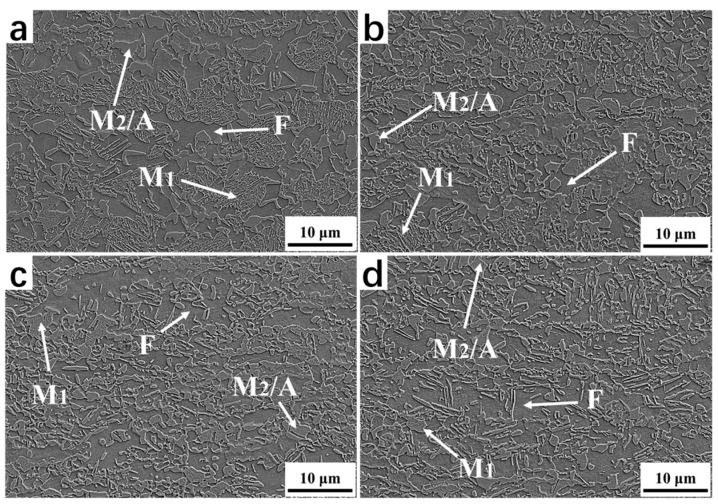
SEM microstructure morphology after the Q&P treatment. (**a**–**d**) Microstructure morphology of 0%, 10%, 40% and 70% cold rolling reduction rates after the Q&P treatment, respectively.

Figure 3 presents the SEM microstructure morphology of the samples after the Q&P treatment, with varying degrees of CRRRs. The morphological features enable the distinction of different phases [26]. Specifically, the ferrite phase is denoted as F; the primary martensite formed during the first quenching and tempered in the partitioning region is denoted as M1; and the secondary martensite/carbon-enriched retained austenite (RA) region is denoted as the M2/A island. The second martensite phase was formed during the second quenching to room temperature. Due to the carbon depletion and tempering of the M1, the surface exhibits rough morphology upon etching. Conversely, the M2/A islands possess smooth surfaces. Therefore, a general distinction can be made according to the surface roughness after etching [27,28]. When observing Figure 3, it is evident that, as the CRRR increases, several changes occur in the microstructure. Firstly, the volume fraction of the primary martensite phase (M1) decreases and is accompanied by a reduction in its size. Simultaneously, there is an increase in the volume fraction of the M2/A island region, indicating an elevated presence of the secondary martensite.

**Figure 4:**
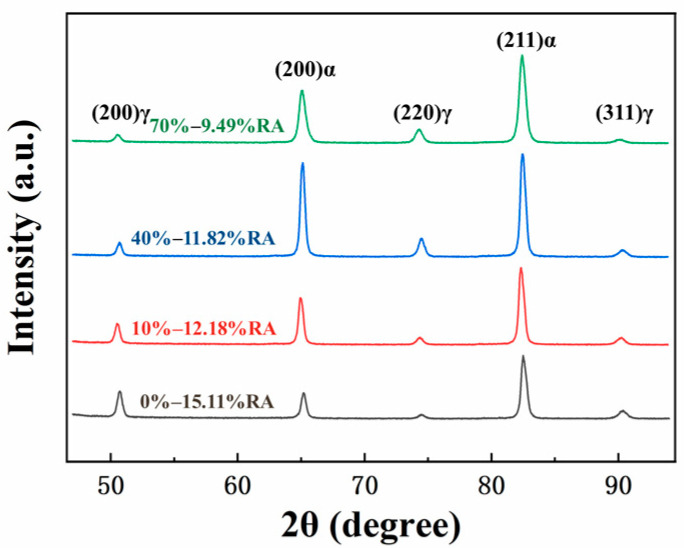
XRD patterns and the corresponding calculated volume fraction of retained austenite for specimens subjected to different cold rolling reduction rates.

Figure 4 showcases the X-ray diffraction (XRD) patterns and the calculated volume fraction of retained austenite (RA) for samples subjected to varying degrees of CRRRs. The analysis of the data reveals a consistent trend: as the CRRR increases from 0% to 70%, there is a gradual and continuous decrease in the volume fraction of retained austenite. Specifically, the volume fraction of retained austenite declines from 15.11% to 9.49% across the range of the CRRRs investigated. These results demonstrate that increasing the CRRR correlates with a reduced presence of retained austenite within the material.

**Figure 5:**
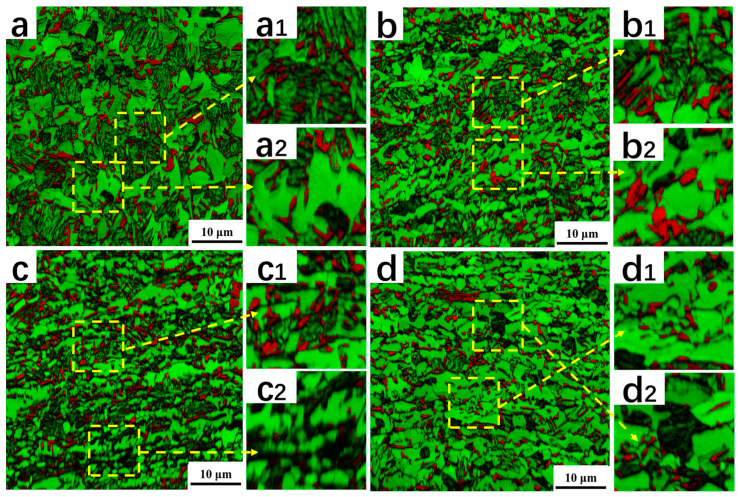
EBSD microstructure morphology after the Q&P treatment (IQ + phase maps). (**a**–**d**) are the microstructure morphology of 0%, 10%, 40% and 70% cold rolling reduction after the Q&P treatment, respectively. Insets denoted with 1 (**a1**–**d1**) are highlighted areas with lath-type retained austenite while insets denoted with 2 (**a2**–**d2**) correlate to secondary martensite (M2).

Figure 5 provides the EBSD results, with retained austenite (RA) shown in red; ferrite with a high IQ in green; tempered martensite (M1) with a poor IQ in grey; and secondary martensite (M2) in dark. The observations were made on specimens with different CRRRs. In the 0% reduction rate sample (Figure 5a), both blocky and lath types of retained austenite were observed. The blocky retained austenite (Figure 5(a2)) is predominantly located within the ferrite phase, while the lath-type retained austenite is observed within the tempered martensite (Figure 5(a1)). The morphology and distribution of retained austenite and martensite are related to the morphology and distribution of austenite in the two-phase zone. Cold rolling leads to the refinement of austenite grains in the two-phase zone, which in turn leads to a reduction in martensite size after cooling so that there is less lath-type retained austenite and more retained austenite in the blocky form at the ferrite grain boundaries or inside the ferrite. With an increase in the CRRR, several changes are noticeable. Firstly, the volume fraction of lath-type retained austenite within the tempered martensite decreases (Figure 5(a1,d1)). Simultaneously, there is an increase in the volume fraction of the secondary martensite (Figure 5(a2,d2)). Additionally, it can be observed that, compared to the 70% reduction rate sample, the ferrite grain size in the 10% and 40% reduction rate samples appears more uneven. This uneven grain size may be attributed to insufficient recrystallization. For a comprehensive overview of the phase distribution, Table 1 provides the exact volume fractions of each phase in the investigated steels.

### 3.3. Mechanical Properties

**Figure 6:**
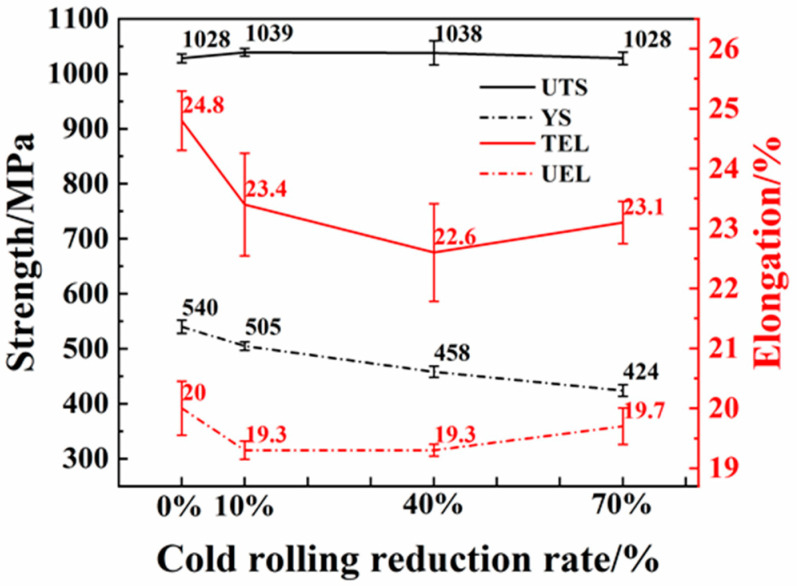
The mechanical properties, including tensile strength, yield strength, uniform elongation and total elongation, were analyzed in relation to the reduction rates of cold rolling.

Figure 6 presents the mechanical properties of Q&P samples with varying CRRRs. The effects of cold rolling on the mechanical properties of the samples are evident from the data. Overall, it is observed that cold rolling has an adverse impact on the mechanical properties of Q&P samples. As the CRRR increases from 0% to 70%, the ultimate tensile strength (UTS) remains relatively stable at approximately 1030 MPa. However, the yield strength (YS) experiences a continuous decrease, declining from 540 MPa to 424 MPa. Furthermore, the uniform elongation (UEL), total elongation (TEL) and necking-zone elongation (NEL) initially decrease as the CRRR increases. However, within a small range, these elongation parameters exhibit a subsequent increase.

**Figure 7:**
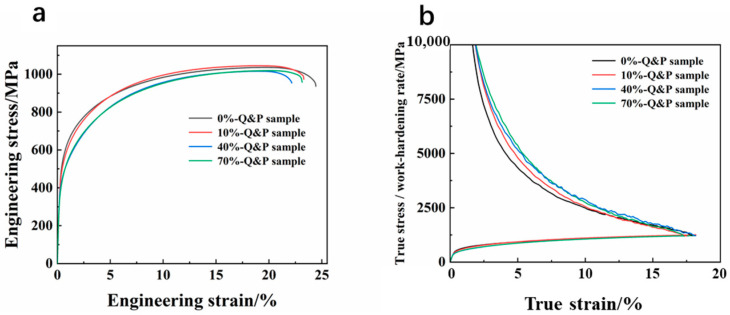
(**a**) Typical stress-strain curves of samples with different cold rolling reduction; (**b**) true stress and work-hardening rate of the corresponding samples.

Figure 7a presents the typical engineering stress-strain curves for different Q&P samples, while Figure 7b displays the true stress and work-hardening rate for the respective samples. Additionally, Table 2 provides a comprehensive summary of the mechanical properties, including tensile strength, yield strength, uniform elongation and total elongation for each Q&P sample. The stress-strain curves in Figure 7a demonstrate variations in the mechanical behavior of Q&P samples. It is observed that different samples exhibit distinct stress-strain responses, indicating differences in their mechanical properties. Notably, there are significant differences in the initial work-hardening rates among the different samples in Figure 7b. As the strain increases, the work-hardening rates decrease. These findings suggest that Q&P samples display diverse mechanical properties, as evident from their stress-strain curves, true stress and work-hardening rates. The variations in the initial work-hardening rates indicate different levels of strain hardening behavior among the samples.

## 4. Discussion

### 4.1. Effect of Cold Rolling on the Microstructures’ Evolution

**Figure 8:**
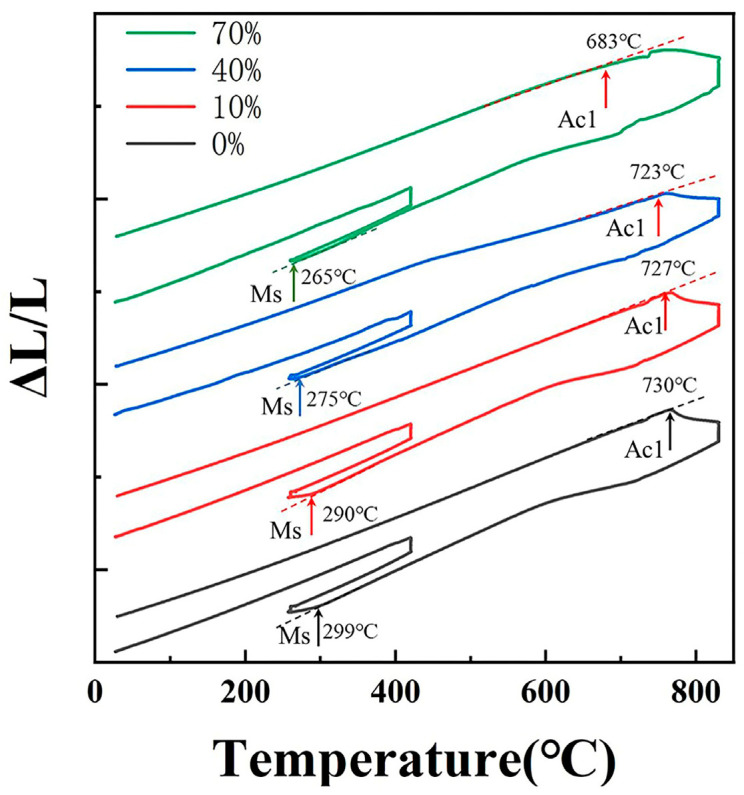
Thermal expansion curve of the Q&P process.

The organizational evolution process in the Q&P (quenching and partitioning) process, particularly the changes in ferrite and retained austenite content, can be understood by studying the thermal expansion behavior of the material. Figure 8 depicts the thermal expansion curve, which provides insights into the phase transition process occurring during Q&P. As the CRRR increases, the temperature of Ac1, representing the start of ferrite-to-austenite transformation, decreases continuously. This phenomenon can be attributed to the increased driving force for phase transformation due to the introduction of deformation energy storage through cold rolling [29]. The reduction in Ac1 temperature indicates a higher tendency for austenite formation with increasing CRRRs. During the uniform cooling process from 830 °C to 710 °C, a change in the slope of the expansion curve is observed, indicating the formation of ferrite. This transition is likely associated with the thermal expansion behavior of the material. The Ms point temperature also decreases with the increase of CRRR which is related to the austenite grain size in the intercritical two-phase region [20,30]. At 420 °C, a significant change in the slope of the expansion curve is observed, indicating the occurrence of bainite transformation in addition to carbon partitioning from martensite to austenite [31]. The bainite phase transition becomes more prominent as the CRRR increases, as evidenced by the increased amount of expansion. Overall, the thermal expansion experiments provide valuable insights into the phase transition processes occurring during the Q&P process. The observed changes in the expansion curve, such as the shift in Ac1 temperature and the distinct slopes, indicate variations in the formation of ferrite and bainite, which correlate with the CRRRs. The increased expansion suggests a higher amount of bainite formation with increasing CRRRs.

**Figure 9:**
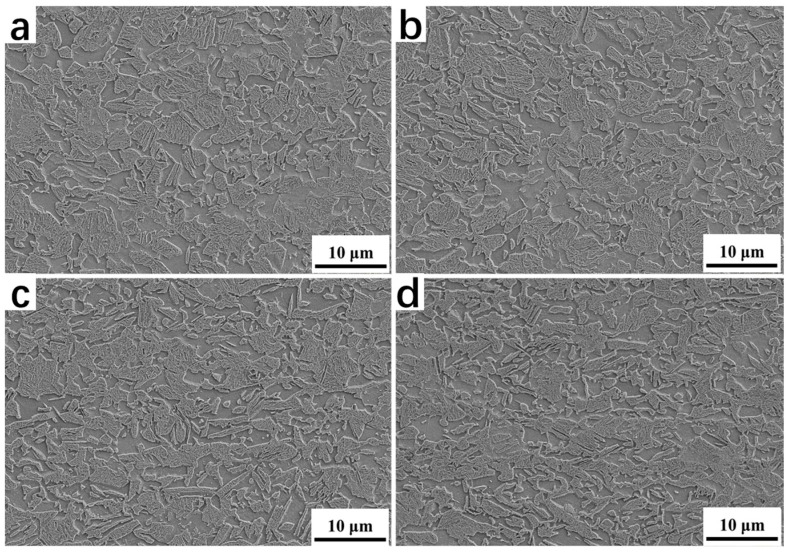
(**a**–**d**) The microstructure morphology of investigated steels treated at 830 °C/100 s for different cold rolling reduction rates of 0%, 10%, 40% and 70%, respectively.

Figure 9 depicts the microstructure at 830 °C/100 s, revealing a decrease in the size of austenite grains with increasing CRRRs. This observation can be attributed to the higher number of nucleation points present in the cold-rolled samples during the austenite transformation process [29,32]. As a result, the size of the austenite grains at high temperatures is reduced. The reduction in austenite grain size at high temperatures has implications for the hardenability of austenite. A smaller grain size decreases the hardenability, resulting in a higher transformation to ferrite during the cooling process [14]. This is the primary reason for the increase in ferrite content with increasing CRRRs. Another contributing factor to the increase in ferrite content is the increased bainitic transformation, which leads to the formation of a bainite-ferrite phase. The decrease in high-temperature austenite grain size results in a decrease in Ms [20,30]. When quenched to the same temperature, the reduction in martensite content leaves insufficient carbon to partition into the retained austenite. As a result, bainitic phase transformation or the formation of martensite occurs during quenching. This reduction in retained austenite content is primarily attributed to these factors.

### 4.2. The Relationship between Microstructure and Mechanical Properties

The mechanical properties of Q&P steel are influenced by various factors, including the volume fraction, morphology, distribution and grain size of each phase. Among these factors, the presence of retained austenite plays a particularly important role [33,34]. During plastic deformation, retained austenite undergoes a strain-induced martensite, known as the transformation-induced plasticity (TRIP) effect. This transformation reduces local stress concentration and delays microcrack formation [35,36,37]; thus, improving the material’s elongation [34,35,36,37,38,39]. In addition, soft-phase ferrite bears more strain leading to yielding of the material, while hard-phase martensite bears more stress. In the investigated Q&P steels, the CRRR affects the volume fraction of retained austenite. As the reduction rate increases from 0% to 70%, the volume fraction of retained austenite decreases from 15.11% to 9.49%, leading to a decrease in elongation. However, it is noteworthy that at a reduction rate of 70%, a slight increase in elongation is observed. This can be attributed to a higher proportion of the soft phase ferrite, which contributes more to the plasticity of the material [40,41]. Furthermore, the distribution of retained austenite within the matrix is also influenced by the CRRR. As the reduction rate decreases, the amount of retained austenite located inside the martensite increases. These retained austenite regions exhibit high stability and effectively inhibit crack propagation at the crack tip after necking; thus, improving elongation during non-uniform deformation stages.

Q&P steels investigated in this study consist of martensite, ferrite, retained austenite and a small amount of bainite. The yield strength of multiphase materials is primarily determined by the soft phase [40,41]. In this case, as the CRRR increases from 0% to 70%, the volume fraction of the soft phase ferrite increases from 27% to 46%, leading to a decrease in yield strength. In summary, the mechanical properties of Q&P steel are influenced by various factors, including the volume fraction and distribution of retained austenite, as well as the presence of the soft phase ferrite. The decrease in elongation with increasing CRRRs is mainly attributed to the decrease in the volume fraction of retained austenite. However, the presence of ferrite in higher proportions at a reduction rate of 70% contributes to a slight increase in elongation. The yield strength is primarily determined by the soft phase, with the increase in ferrite volume fraction leading to a decrease in yield strength as the CRRR increases.

The TSCR process holds significant promise for Q&P steel production due to its distinct advantages over conventional hot-rolling methods. Specifically, the shorter line lengths and smaller investments of TSCR, in comparison to the traditional hot rolling processes, makes it an attractive choice for industrial applications. These factors contribute to enhanced operational efficiency and reduced production costs. Moreover, in terms of environmental sustainability and carbon neutrality, the TSCR process exhibits lower carbon emissions throughout the production chain; thus, resonating with the automotive industry’s emphasis on “green” and low-carbon materials throughout their life cycle. As the automotive industry increasingly places value on sustainable practices, the TSCR process stands out as a desirable solution that can address these environmental priorities.

## 5. Conclusions

This study examined the influence of the cold rolling reduction rate on the microstructure and properties of Q&P steel with a ferrite-pearlite initial structure. Based on the findings, the following conclusions can be drawn:

(1) The mechanical properties of Q&P steel are adversely affected by cold rolling, resulting in a reduction in both yield strength and plasticity;
(2) The decrease in yield strength is primarily attributed to the increase in ferrite content, which serves as the soft phase in the multiphase material. As the cold rolling reduction rate increases, the volume fraction of ferrite increases, leading to a decrease in yield strength;
(3) The decrease in plasticity is mainly influenced by the reduction in retained austenite content. As the cold rolling reduction rate increases, the volume fraction of retained austenite decreases, which results in a decrease in plasticity;
(4) It is recommended that, when employing the TSCR process for the production of Q&P steels, the necessary thickness required for the application should be directly achieved through hot rolling.

## Figures and Tables

**Table 1:** Volume fraction of each phase of investigated steels under different cold rolling reduction rates.

Cold Reduction	0%	10%	40%	70%
Ferrite/%	27.60 ± 1.83	34.70 ± 1.99	39.30 ± 2.63	46.10 ± 1.59
Martensite/%	57.29 ± 2.17	53.12 ± 2.44	48.88 ± 3.06	44.41 ± 1.82
Retained austenite/%	15.11 ± 0.34	12.18 ± 0.45	11.82 ± 0.43	9.49 ± 0.23

**Table 2:** Mechanical properties of investigated steels. YS is the yield strength, UTS is the ultimate tensile strength, UEL is the uniform elongation, TEL is the total elongation, and NEL is the necking-zone elongation of each sample.

Cold Reduction	0%	10%	40%	70%
YS/MPa	540 ± 12	505 ± 8	458 ± 10	424 ± 10
UTS/MPa	1028 ± 8	1039 ± 7	1038 ± 22	1028 ± 11
UEL/%	20.0 ± 0.5	19.3 ± 0.2	19.3 ± 0.1	19.7 ± 0.3
TEL/%	24.8 ± 0.5	23.4 ± 0.9	22.6 ± 0.8	23.1 ± 0.4

## Data Availability

Not applicable.
